# Predisposing, Enabling, and Need Factors Associated with the Choice of Pharmacy Type in the US: Findings from the 2015/2016 National Consumer Survey on the Medication Experience and Pharmacists’ Roles

**DOI:** 10.3390/pharmacy9020072

**Published:** 2021-03-28

**Authors:** Mohamed Rashrash, Suhila Sawesi, Jon C. Schommer, Lawrence M. Brown

**Affiliations:** 1 Department of Pharmaceutical and Administrative Sciences, University of Charleston School of Pharmacy, Charleston, WV 25304, USA; 2 Department of Public Health and Community Medicine, Tufts University School of Medicine, Boston, MA 02155, USA; Suhila.sawesi@tufts.edu; 3 Department of Pharmaceutical Care & Health Systems, University of Minnesota Twin Cities College of Pharmacy, Minneapolis, MN 55455, USA; schom010@umn.edu; 4 Department of Biomedical and Pharmaceutical Sciences, School of Pharmacy, Chapman University, Irvine, CA 92866, USA; lbbrown@chapman.edu

**Keywords:** Anderson model, pharmacy selection, pharmacy choice, type of pharmacy, pharmacy patronage

## Abstract

Background: Knowing the type of pharmacy used by the patient is meaningful to the pharmacist. Previous studies have assessed different factors predicting the kind of pharmacy selection and reached inconsistent findings. Objectives: To identify patient and health-related factors associated with pharmacy type selection. Methods: The Andersen Behavioral Model of Health Service Use was used to organize the selection of patient characteristics and categorize them as predisposing, enabling, and need factors. The dependent variable was the type of pharmacy used. Logistic regression was used to predict the association between patient-related characteristics and the type of pharmacy used. Results: Older age respondents were less likely to use independent pharmacies (OR = 0.992) and more likely to use mail pharmacy services (OR = 1.026). Highly educated people showed higher use of chain and mail pharmacies (OR = 1.272, 1.185, respectively) and less tendency to use the independent, supermarket, and prescription-only pharmacy types. Men were less likely to use chain pharmacies (OR = 0.932) and more likely to use supermarket pharmacies than women. Patients who use Medication Therapy Management (MTM) services had higher odds of using independent and supermarket pharmacies (OR = 2.808, 1.689, respectively). Patients with a higher number of chronic diseases and experienced side effects of medications were more likely to use independent pharmacies (OR for number of disease = 1.097 and for side effects = 1.095). Conclusions: This study’s findings identify characteristics associated with selecting certain pharmacy settings and direct future research to include other predictors encompassing beliefs, attitudes, and other social factors.

## 1. Introduction

Knowing the type of pharmacy used by the patient is important to the pharmacist. Previous studies found that a pharmacy’s location and convenience to the patient’s residence and work, in addition to medical providers’ location, were the most critical factors affecting pharmacy type selection [1,2,3,4,5,6]. The importance of factors affecting the selection of pharmacy is still under debate among researchers and experts. The influence of these factors is a debatable subject and needs more research [7,8]. Patient preferences in selecting a pharmacy type are more similar to preferences when selecting grocery stores than choosing non-pharmacist medical providers [9]. Many factors affect the choice of the kind of pharmacy, including the following: experienced pharmacists, convenience, branded pharmacies, and demographic variables [10,11,12,13,14].

Demographic factors, such as age, occupation, and others, may influence pharmacy consumers’ buying decisions. The demographic characteristics are significant factors associated with the store type selected by patients [15]. Convenience is the most important factor affecting the choice of pharmacy type by a younger population. On the other hand, retired customers with moderate to low income highly value pharmacy services. They show higher loyalty levels to individual pharmacies, and they look to more personal relationships with pharmacists. These indicate the possible effects of demographic characteristics, such as age on pharmacy selection by different customers [16].

### Anderson Model

Patients need to meet with health care professionals at a certain point, and the process is called health care utilization (HCU). Research has shown that HCU might be dependent on the social characteristics of patients. For example, women utilize health care services more frequently than men. Scientists have developed frameworks to determine predictors of HCU [17]. The most well-known framework in the prediction of HCU is the Behavioral Model of Health Services Use. This model was developed by Ronald M. Andersen in 1968 [18,19,20,21,22,23,24,25,26].

The model incorporates individual and contextual factors of HCU. It divides contextual determinants in the same way as individual determinants have been separated to predispose, enable, and need to utilize health service [25]. Andersen and Davidson [19] recently explained these factors as follows: predisposing factors include demographic factors such as age and gender [19], and social characteristics encompass education, job, ethnicity, marital status, and mental determinants such as health beliefs, attitudes, values, and knowledge regarding health services and general health. Enabling factors involve financial determinants and factors related to the health care system. Individual economic determinants include annual income and the savings that make an individual capable of paying for health care services, determined by an individual’s health insurance status. Organizational-related factors include the amount, structure, and distribution of health services personnel and facilities. Health policies, office hours, and physician density are also considered enabling factors. Need factors as proposed by Andersen and Davidson [19] distinguish between the perceived need for health services including perceived general health, functional status, and symptoms in addition to the evaluated need (assessment of the need for medical care by professionals). They also differentiate between environmental need factors and population health indices. The environmental factors involve health conditions related to the environment (work, traffic, and crime-related injuries and deaths), while population health indices apply epidemiological indicators such as mortality and morbidity, which is considered an overall measure of health of the community.

This study was to compare factors that influence consumers’ selection of pharmacy type in the USA using the Anderson Model as a framework. Additionally, the study was to assess the relationships between predisposing, enabling, and need factors and pharmacy type used by consumers. The study can answer the following question: Which factors associated with or which predict the pharmacy type are used by the study population?

## 2. Method

### 2.1. Data Source

This study used the 2015 National Consumer Survey on the Medication Experience and Pharmacist Role (NCSME-PR) as the only data source. The NCSME-PR is a national cross-sectional, online survey used to collect pharmacy consumers’ data with technical support from Qualtrics Panels. The data encompassed responses from 50 US states and the District of Columbia, with a total sample of 26,174 respondents. The Qualtrics panel emailed the survey to the target population. All duplicated responses were discarded by applying a unique identifier. Samples developed by Qualtrics matched the census statistics for geographic location, age, and gender. The inclusion criteria were any US resident of 18 years of age and older and showed readiness to take the survey. The Human Subjects Committee at the University of Minnesota exempted Institutional Review Board (IRB) approval.

### 2.2. Study Variables

#### 2.2.1. The Dependent Variable

The dependent variable was collected using a question about the type of pharmacy used by the consumer to get their medications (“If you need a prescription medication today, what type of pharmacy would you use for obtaining it?”). The question did not ask how many times they use the pharmacy in a time frame such as last week, month, or year. The variable is categorized into five categories: independent pharmacy, chain pharmacies such as CVS and Walgreens, supermarket and mass merchandise pharmacies such as Wal-Mart and Kroger, mail order pharmacy, and finally prescription-only pharmacy, which mainly include Veteran (VA) hospital, and clinic pharmacies.

#### 2.2.2. Independent Variables

Variables were divided using the Andersen Behavioral Model of Health Care Utilization, which includes three factors: predisposing factors, enabling factors, and need factors. This method has been previously applied in pharmacy-related research by many researchers [27,28].

Predisposing factors included demographic factors such as age, gender, education, race, and residence region. Age was ordered into categories: 18 to 33 (born between 1982 and 1997 for this study), 34 to 50 (born between 1965 and 1981), 51 to 69 (born between 1946 and 1964), and 70 or older (born before 1945). The race involved five groups (Native American, Latino/Latina, Asian, White, Black/African American, Other)**.** White was the primary race, so all other races were merged into only one category called non-white. Regions were categorized into North East, Midwest, South, and West. Education was categorized into two categories (high school and less and college degree and higher).

Enabling factors encompass income, medical insurance, medication insurance, and mail order pharmacy use. Income was categorized into 3 categories ($40,000 or less, $41,000 to $65,000, and more than $65,000). Health insurances were categorized into insured and uninsured. The mail order variable was categorized as use and not use.

Need factors involved perceived health status, use of prescription, herbal, and OTC medications, number of medical conditions. Perceived health status was categorized as excellent, good, fair, and poor. The total number of chronic diseases was determined by asking the question: “How many health problems do you currently have?”. Other factors were the use of pharmacy-based vaccination, Medication Therapy management (MTM) services, previous side effect episodes, and use of drive-through services.

### 2.3. Data Analysis

Data analysis was conducted using SPSS version 22. Descriptive statistics were computed for all included variables in the study. Logistic regression analysis was used to find any relationship between predisposing, enabling, or need factors and the type of pharmacy being used by respondents

## 3. Results

Characteristics of the study population are described in Table 1. The study sample was 26,174 respondents with a mean age of our study population were 44.21 ± 16.55.

Predisposing factors, as shown in Table 1, reveal that the sample mainly comprised white participants, as more than three-quarters of respondents were white. Further, more than half of the study population had less than a college degree as their level of education. The sample was divided into four regions, with the highest proportion from the South, while the North East region had the lowest representation. Female gender was the predominant gender in the study population (71.2%), with the main age groups of 18–33 year and 51–69 year (32.6, 30%, respectively).

On the other hand, enabling factors showed that about 82% of the sample individuals had medication or medical insurance. Sample individuals were mainly of low income, as 55.5% earned less than $44,000 annually, and only 20% earned more than $65,000. About half of them reported experiencing financial difficulties (46.6%).

Need factors showed that more than half were taking OTC and prescription medications (53.4%, 64%, respectively). About one-third reported buying herbal drugs and receiving a vaccination at the pharmacy (35.1%, 30.8%, respectively). MTM was among the most used services provided by the pharmacy (2.8%). A total of 26.4% reported side effects experienced from the medications they were taking. The majority of respondents (81.8%) rated their health as either good or fair, and a low proportion reported it as poor (4.1%). Finally, the primary type of pharmacies used were chain and supermarket/mass merchandise pharmacies (40.8%, 34.7%, respectively), with the lowest proportion using mail pharmacies (4.5%).

Table 2 describes the different pharmacies’ predictors with the adjusted odds ratios (OR) from logistic regression analyses. Several factors showed a significant prediction of the use of the type of pharmacy. Being white showed a significant prediction of using various kinds of pharmacies. The findings indicated a higher use of independent pharmacies, supermarket/mass merchandise, and mail pharmacies with less use of chain and prescription-only pharmacies. Older respondents were less likely to use independent pharmacies (OR = 0.992) and more likely to use mail pharmacy services (OR = 1.026). Highly educated people showed higher use of chain and mail pharmacies (OR = 1.272, 1.185, respectively) and less tendency to use independent, supermarket/mass merchandise, and prescription-only pharmacies types. The geographic region had no relationship with the kind of pharmacy used. Men were less likely to use chain pharmacies (OR = 0.932) and more likely to use supermarket/mass merchandise pharmacies than women. Categorizing by age showed that the 18–33 age category was less likely to use independent pharmacies and more likely to use other pharmacies than their older counterparts (OR = 0.805, 1.228 consecutively). Additionally, the 51–69 age group was less likely to use prescription-only pharmacies (OR = 0.558). A number of people using prescription-only pharmacies were receiving Veteran Benefits, and therefore, they must use the VA system. Hence, demographic characteristics were not the determinant factors in their selection of pharmacy type.

Enabling factors showed several significant relationships with the type of pharmacy being used by study participants. People with medication insurance showed different magnitudes of utilization when considering the value of OR to measure the strength of relationships between dependent and independent variables. People with medication insurance showed higher odds of using chain pharmacies and supermarket/mass merchandise pharmacies compared to others (OR = 0.833, 0.744, respectively). Consumers who had previously used mail pharmacies showed higher odds of using prescription-only pharmacies; however, they were less likely to use independent, chain, and supermarket/mass merchandise pharmacies (OR = 0.705, 0.538, 0.560 correspondingly). Income showed no significant relationship with the usage of pharmacies. Individuals passing through financial hardships had fewer odds of using chain (OR = 0.862) and prescription-only pharmacies (OR = 0.770) but showed higher odds of using supermarket/mass merchandise pharmacies (OR = 1.245).

The analyses showed that need factors have several significant relationships with the type of pharmacy used by respondents. People who were using OTC medication were less likely to use mail pharmacies (OR = 0.751) and prescription-only pharmacies (OR = 0.890), but they were more likely to use supermarket/mass merchandise pharmacies (OR = 1.13). Further, herbal drug users were more likely to use supermarket/mass merchandise pharmacies than other types (OR = 1.082) and had fewer odds of using mail pharmacies (OR = 0.839). Respondents who received vaccinations provided by pharmacies were more likely to use chain pharmacies (OR = 1.13) and less likely to use prescription-only pharmacies (OR = 0.770).

Patients who used MTM services had higher odds of using independent and supermarket/mass merchandise pharmacies (OR = 2.808, 1.689, respectively). Respondents who reported using drive-through services were most likely to use chain pharmacies (OR = 3.282). Individuals with a higher number of chronic diseases and who experienced side effects of medications were more likely to use independent and prescription-only pharmacies (OR for number of diseases = 1.097, 1.058 and for side effects = 1.095, 1.165, respectively).

Finally, rated health was a significant factor predicting the use of pharmacy type. People who rated their health as good or fair compared to poor were more likely to use chain pharmacies (OR = 1.167, 1.184 consecutively), while all rated health categories were less likely to use independent pharmacies than people who rated their health as poor (OR = 0.738, 0.835, 0.836 correspondingly)

## 4. Discussion

Previous research has studied the demographic and population characteristics directly related to the consumers’ selection of pharmacy type. Age was of essential factors that were directly related to the choice of pharmacy type [29,30,31].

Marital status and insurance coverage were not significant in this study, and these findings are consistent with the results of previous research [31,32,33]. Other factors have shown mixed results. Previous studies found that gender, level of education, income, and health-related purchases such as OTC medication and herbal medicines showed a significant relationship with the type of pharmacy used by consumers [4,32,34]. However, some research findings did not show any relationships with these factors [4,30,33].

Inconsistencies in these findings between different studies may be due to differences in the data collection process. Some of these data were collected by phone and some by personal interviews or distribution through the mail and use different sampling techniques from random to convenient sampling [2]. Moreover, the data were collected in different periods and various locations; findings may differ because of variations among consumer groups [31].

In one previous study, scientists found significant relationships between demographic factors and the choice of chain and independent pharmacies, which is congruent with this study’s findings [30]. Independent pharmacy consumers were older and had drug insurance covering their prescriptions. This conclusion agrees with our findings that older patients prefer independent pharmacies, which may be attributed to pharmacists’ friendliness and professionalism in providing their services [31].

Generally, previous research findings revealed that younger consumers like to use chain pharmacies, while older counterparts favor independent pharmacies. This study found that younger consumers (18–33 y) were less likely to use independent pharmacies than older consumers (70 or older). There was no association between being middle-aged (34–50 y) and selection of the type of pharmacy.

Education was a significant predictor of the type of pharmacy used, which is the same finding as Lipowski [31] and contrary to Carroll and Jowdy’s [30] findings. Highly educated people tended to use chain and mail pharmacies, and these results are consistent with other research findings [35] and not compatible with Carroll and Jowdy’s conclusions. The male gender showed a significant positive relationship with supermarket and mass merchandise pharmacy use and a negative association with chain pharmacy use.

Enabling factors were statistically significant variables except for income. Consistent with the finding of Carroll and Jowdy [30], and contrary to the conclusions from Lipowski [31], the availability of insurance coverage for prescription drugs was significant in all types of pharmacy chosen. However, certain factors from the Anderson Model were significant predictors of pharmacy selection. The model still lacks explanatory power because some crucial factors were not in the model. Attitudes about pharmacy and other attributes were significant predictors as found by Caroll and Jowdy [30]. Other studies also found that attitude and belief were essential factors in predicting consumer selection of services, followed by demographic and psychosocial factors [36].

## 5. Limitations

This study had several limitations. First, insurance may mandate the use of specific pharmacies, which will affect the choice of pharmacy type used by consumers in certain cases. This limitation could be considered if the questionnaire asked the respondents about their medication insurance’s name and type. Second, because our data were self-reported, recall bias can be expected. This bias may lead to false reporting of the kind of pharmacies used by respondents. Third, this study used an electronic survey, which might be vulnerable to participants’ selection bias as internet nonusers were not included in this study. Fourth, the categorization of prescription-only pharmacies had many types of pharmacies that might give a better view of predictors if it was broken down to more pharmacy channels such as hospitals, clinics, and VA pharmacies. Fifth, other factors not included, such as attitude and belief, were not examined because the study was a secondary analysis, and there was no way to ask for them. Finally, the questionnaire did not include questions regarding the rationale for pharmacy type choice, whether related to economic or quality of service or other reasons.

## 6. Conclusions

This study’s findings identified characteristics associated with the selection of certain pharmacy settings. All three subscales of the Anderson model showed a significant prediction of the type of pharmacy chosen by the consumers. Predisposing factor analysis revealed that race and education were the strongest predictors of the pharmacy type selection. Enabling factors also showed a significant prediction of pharmacy channel selection except for the income, which was insignificant with all pharmacy types. All need factors showed significant relationships with the choice of pharmacy type. Choice of type of pharmacy used by consumers is a complex decision affected by many factors. Pharmacists need to know the factors influencing consumers’ choices, especially those of younger ones and people without health insurance.

## 7. Future Research

Logistic regression analysis using the Anderson Model helps to assess factors associated with choosing the type of pharmacy used by consumers. Future research should include attitude and beliefs and more variables such as family size, job, monthly purchase, and self-paid expenses. Additionally, longitudinal research may be required to follow changes in consumer choices over time and as people advancing in age.

## Figures and Tables

**Table 1:** Characteristics of the study population.

Patient Characteristics	Categories	Frequency	Percentage
Predisposing Factors			
Race/Ethnicity	Non-White	5048	19.3
	White	21,125	80.7
Education	Less than bachelor’s degree	16,487	63.0
	Bachelor’s degree or Higher	9686	37.0
Division Region	North East	4617	17.6
	Midwest	6158	23.5
	South	8722	33.3
	West	6670	25.5
Sex	Female	18,625	71.2
	Male	7548	28.8
Age	Age 18–33	8532	32.6
	Age 34–50	1799	6.9
	Age 51–69	7847	30.0
	Age 70 or older	1799	6.9
**Enabling Factors**			
Drug Insurance	Yes	21,444	81.9
	No	4729	18.1
Received prescription through mail	no	21,724	83.0
	yes	4449	17.0
Income	$40,000 or less	11,635	44.5
	$41,000 to $65,000	9140	34.9
	More than $65,000	5398	20.6
Financial Hardship	Yes	12,202	46.6
Medical Insurance	Not covered	4729	18.1
	Covered	21,444	81.9
**Need Factors**			
Taking OTC medications	Yes	13,967	53.4
Taking Prescription Drugs	Yes	16,738	64.0
Using Herbal Products	Yes	9194	35.1
Received vaccination at pharmacy	Yes	8060	30.8
Used MTM	Yes	739	2.8
Used Drive-Through	Yes	9026	34.5
Side Effects	Yes	6903	26.4
Rated Health	Excellent	3679	14.1
	Good	14,647	56.0
	Fair	6762	25.8
	Poor	1085	4.1
Type of Pharmacy	Independent Pharmacy	3422	13.1
	Chain Pharmacy	10,682	40.8
	Super Market/Mass Merchandise	9086	34.7
	Mail Pharmacy	1169	4.5
	Prescription-only Pharmacy	1814	6.9

**Table 2:** Logistic regression analysis of patient characteristics and predictors of the type of pharmacy used.

Patient Characteristics	Independent		Chain		Super/Mass		Mail		Prescription- only	
	Sig	OR	Sig	OR	Sig	OR	Sig	OR	Sig	
**Predisposing Factors**										
White	0.000	1.277	0.000	0.805	0.000	1.309	0.060	1.217	0.000	0.558
AGE	0.027	0.992	0.495	0.998	0.859	1.000	0.000	1.026	0.218	1.006
Bachelor or more	0.000	0.736	0.000	1.27	0.011	0.931	0.016	1.185	0.037	0.896
Division Region	0.249	0.980	0.525	1.01	0.749	0.996	0.712	1.012	0.282	1.026
Male	0.119	1.065	0.007	0.923	0.012	1.077	0.886	0.989	0.185	0.929
Age 70 or older	Ref		Ref		Ref		Ref		Ref	
Age 18–33	0.004	0.805	0.913	1.006	0.905	0.994	0.532	1.106	0.042	1.228
Age 34–50	0.055	1.328	0.707	1.041	0.047	0.808	0.490	0.844	0.759	0.943
Age 51–69	0.215	1.108	0.971	1.002	0.325	0.942	0.923	0.985	0.000	0.558
**Enabling Factors**										
Drug Insurance	0.000	0.449	0.009	0.844	0.000	0.733	0.000	0.042	0.000	0.297
Used prescription mail	0.000	0.705	0.000	0.538	0.000	0.560	0.000	73.746	0.000	1.932
Income	0.507	1.016	0.431	0.986	0.914	0.998	0.456	0.967	0.213	1.041
Financial Hardship	0.051	1.079	0.000	0.862	0.000	1.245	0.058	0.870	0.000	0.770
Medical Insurance	0.000	0.434	0.045	0.875	0.000	0.703	0.000	0.038	0.000	0.333
**Need Factors**										
Use OTC	0.741	0.987	0.118	0.956	0.000	1.130	0.000	0.751	0.029	0.890
Use Herbal	0.262	0.956	0.829	0.994	0.005	1.082	0.015	0.839	0.270	0.943
Vaccinated at Pharmacy	0.156	0.942	0.000	1.130	0.931	1.003	0.327	0.932	0.000	0.770
Used MTM	0.000	2.808	0.000	0.385	0.000	1.689	0.000	0.426	0.064	1.302
Used Drive-Through	0.000	0.809	0.000	3.282	0.000	0.423	0.000	0.514	0.000	0.375
No of Diseases	0.000	1.097	0.000	0.948	0.583	1.007	0.000	0.895	0.011	1.058
Side Effects	0.034	1.095	0.144	0.955	0.672	0.987	0.213	0.903	0.008	1.165
Rated Health Poor	Ref		Ref		Ref		Ref		Ref	
Rated Health Excellent	0.003	0.738	0.104	1.131	0.183	1.105	0.979	0.677	0.240	0.852
Rated Health Good	0.045	0.835	0.024	1.167	0.748	1.022	0.843	0.602	0.334	0.888
Rated Health Fair	0.054	0.836	0.017	1.184	1.000	1.000	0.857	0.605	0.354	0.889

## Data Availability

Data sharing is not applicable to this article.
